# A Self-Attention Legendre Graph Convolution Network for Rotating Machinery Fault Diagnosis

**DOI:** 10.3390/s24175475

**Published:** 2024-08-23

**Authors:** Jiancheng Ma, Jinying Huang, Siyuan Liu, Jia Luo, Licheng Jing

**Affiliations:** 1 School of Computer Science and Technology, North University of China, Taiyuan 030051, China; 2 School of Mechanical Engineering, North University of China, Taiyuan 030051, China; 3 School of Energy and Power Engineering, North University of China, Taiyuan 030051, China

**Keywords:** graph convolutional network, fault diagnosis, rotating machinery, Legendre polynomial, graph theory

## Abstract

Rotating machinery is widely used in modern industrial systems, and its health status can directly impact the operation of the entire system. Timely and accurate diagnosis of rotating machinery faults is crucial for ensuring production safety, reducing economic losses, and improving efficiency. Traditional deep learning methods can only extract features from the vertices of the input data, thereby overlooking the information contained in the relationships between vertices. This paper proposes a Legendre graph convolutional network (LGCN) integrated with a self-attention graph pooling method, which is applied to fault diagnosis of rotating machinery. The SA-LGCN model converts vibration signals from Euclidean space into graph signals in non-Euclidean space, employing a fast local spectral filter based on Legendre polynomials and a self-attention graph pooling method, significantly improving the model’s stability and computational efficiency. By applying the proposed method to 10 different planetary gearbox fault tasks, we verify that it offers significant advantages in fault diagnosis accuracy and load adaptability under various working conditions.

## 1. Introduction

Rotating machinery plays an indispensable role in modern industrial systems and is extensively utilized in industries such as aerospace, automotive, and wind power generation [1]. When operating under complex and harsh conditions such as heavy loads, high temperatures, and high speeds, rotating machinery inevitably experiences various types of faults [2]. The reliable operation of these mechanical devices is crucial to production efficiency, safety, and energy utilization efficiency. Therefore, timely and accurate diagnosis of rotating machinery faults is essential for ensuring production safety, reducing economic losses, and improving efficiency. Fault diagnosis can help maintenance personnel implement preventive measures before a fault occurs, thereby extending equipment life, optimizing maintenance and repair plans, and reducing downtime.

However, fault diagnosis of rotating machinery encounters numerous challenges in practical engineering applications, including the high dimensionality, non-linearity, and non-stationarity of the data, as well as the diversity of fault modes and weak signals of early faults. Such factors significantly increase the difficulty of diagnosis. To address these challenges, numerous fault diagnosis methods and techniques have been developed, including traditional signal processing methods, machine learning techniques, deep learning techniques, and recently, emerging graph-neural-network-based methods. Although these methods have shown some progress in improving the accuracy and efficiency of fault diagnosis, they still face limitations in handling complex data structures, achieving early fault detection, and enhancing model generalization capabilities. Therefore, exploring and developing more efficient and intelligent fault diagnosis methods has become an important research direction in this field. In response to this, this paper introduces a fault diagnosis method for rotating machinery based on graph convolutional neural networks.

Benefiting from advances in deep learning theory, researchers have developed various intelligent fault diagnosis models based on deep learning. These models enable end-to-end fault diagnosis without the need for manual feature extraction. Feng [3] proposed a local connection network (LCN) constructed using a normalized sparse auto-encoder (NSAE) for intelligent fault diagnosis, integrating feature extraction and fault recognition into a general-purpose learning procedure, effectively identifying the health condition of machinery. Chen [4] proposed a novel diagnostic model combining convolutional neural networks (CNN) and extreme learning machines (ELM), achieving higher classification accuracy with less computation time. Jia [5] designed a KMedoids clustering method based on dynamic time warping (DTW-KMedoids) to cluster multi-channel signals, which were then input into clustered blueprint separable convolutions (CBS-Conv) for end-to-end HST bogie fault diagnosis. Zhang et al. [6] proposed a method for identifying types of rotating machinery faults based on recurrent neural networks (RNNs). Shi et al. [7] proposed a multi-scale feature adversarial fusion network for unsupervised cross-domain fault diagnosis. Kong et al. [8] proposed a multi-task self-supervised method to mine fault diagnosis knowledge from unlabeled data. Liu et al. [9] proposed a continuous learning model based on weight space meta-representation (WSMR) for fault diagnosis of switch machine plunger pumps. Quan [10] utilized the IJDA mechanism and I-Softmax loss to construct a deep discriminative transfer learning network (DDTLN) for fault transfer diagnosis.

Intelligent fault diagnosis based on deep learning has yielded numerous results. Traditional deep learning models can only input fixed-dimensional data, and the local input data must be ordered. However, widely used deep learning models such as CNNs struggle to achieve optimal performance in fields involving non-Euclidean structured data due to their inherent structural characteristics [11]. Given the universality of graph structures, extending deep learning to graph structures has garnered increasing attention, leading to the emergence of graph neural networks (GNNs) and the development of models such as GCN [12], GraphSAGE [13], and GAT [14]. These models are widely used in computer vision [15], natural language processing [16], and recommendation systems [17].

Inspired by this, researchers have started to apply GNNs to the field of fault diagnosis. Li [18] proposed a multi-receptive field graph convolutional network (MRF-GCN) and verified its effectiveness in mechanical fault diagnosis. Chen [19] proposed a GCN-based fault diagnosis method based on structural analysis, which converts the collected acoustic signals into association graphs and inputs them into the GCN model to achieve fault diagnosis of rolling bearings. Zhao [20] designed a new multi-scale deep graph convolutional network (MS-DGCN) algorithm to diagnose rotor-bearing system faults under fluctuating conditions. Yang [21] proposed a deep capsule graph convolutional network (DCGCN) method to diagnose compound faults in harmonic drives. Yang [22] proposed a feature extraction method based on spatiotemporal graphs, called SuperGraph, for fault diagnosis of rotating machinery. Li [23] proposed an adaptive multi-channel heterogeneous graph neural network (AMHGNN), which achieves more accurate node classification through flexible topological structures. Chen [24] proposed a neighborhood convolutional graph neural network (NCGNN) that avoids training the model with an adjacency matrix, effectively controlling training costs and enhancing scalability. Wang [25] proposed a novel temporal–spatial graph neural network with an attention-aware module (A-TSGNN) for mechanical fault diagnosis. Zhang [26] proposed a granger causality test-based bearing fault detection graph neural network method (GCT-GNN). Cao [27] proposed a novel pulse graph attention network for intelligent fault diagnosis of planetary gearboxes, achieving simultaneous extraction of spatiotemporal features from gearbox signals. Yu [28] proposed a two-stage importance-aware subgraph convolutional network (I^2^SGCN) based on multi-source sensors, which improves the fault recognition performance of intelligent neural networks under variable conditions and limited data. Zhong [29] designed a hierarchical GCN with latent structure learning (HGCN-LSL) for industrial fault diagnosis. This algorithm organizes hierarchical networks to collaboratively improve the quality of the latent graph structure, thereby ensuring enhanced diagnostic performance.

The contributions of this paper can be summarized as follows:

1. We propose a method for constructing association graphs based on vibration signals, which transforms vibration signals in Euclidean space with translation invariance into graph signals in non-Euclidean space.
2. We propose a fast local spectral filter based on Legendre polynomials. Compared to traditional Chebyshev filters used in graph neural networks, it enhances the model’s stability and load adaptability.
3. We propose a graph pooling method based on self-attention for fault diagnosis of rotating machinery. This method adaptively focuses on key nodes in the graph to effectively capture fault features, thereby improving the accuracy of fault diagnosis.

## 2. Fault Diagnosis Model Based on SA-LGCN

**Figure 1:**
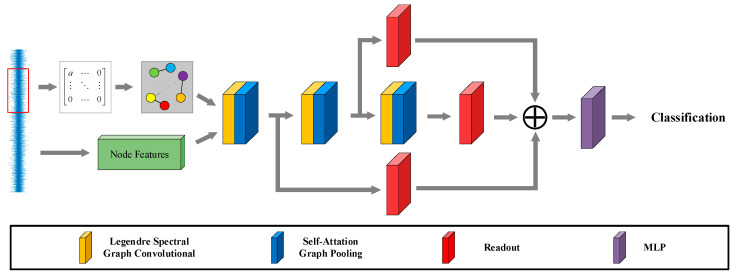
SA-LGCN model.

The collected vibration signals of rotating machinery include both normal and fault vibration signals. By introducing the KNN algorithm, the collected vibration signals are converted into non-Euclidean structured data, which contain more information. However, non-Euclidean data lacks translation invariance, making traditional convolutional neural networks unsuitable. Therefore, this paper utilizes graph convolutional networks (GCNs) to extract the spatial features of the topology graph from non-Euclidean structured data. Consequently, fault diagnosis of rotating machinery is transformed into a graph classification task within the graph convolutional network framework. To address the issue of weak early fault signals in rotating machinery, which are difficult to distinguish from normal signals, this paper proposes a fault diagnosis model based on self-attention pooling and a Legendre graph convolutional neural network, referred to as SA-LGCN. The model comprises four main components: (1) vibration signal association graph construction; (2) Legendre graph convolution; (3) self-attention graph pooling; and (4) readout. In this section, each component of the proposed model will be described in detail. The model is illustrated in Figure 1.

### 2.1. Vibration Signal Association Graph Construction

In practice, the collected vibration signals are typically one-dimensional time-domain signals. By applying the KNN algorithm, these signals are converted into a graph, i.e., transformed from the general data domain to the graph domain. Suppose the length of the collected vibration signal X is L. First, the original data X are divided into a set of sub-samples with a length of d, where the sub-samples are independent and non-overlapping. The process is described as follows:

(1) $$\left\{\begin{array}{l} Y=\left(x_{1},x_{2},x_{3},\ldots ,x_{n}\right),\\ n=\left\lceil \frac{L}{d}\right\rceil ,\\ x_{i}\in X,x_{i}\cap x_{j}=\emptyset ,\\ i,j\in \left\{1,2,3,\ldots ,n\right\}, \end{array}\right.$$

where Y represents the set of sub-samples, n represents the number of sub-samples, and $\left\lceil \cdot \right\rceil$ represents the ceiling operator. Each sub-sample $x_{i}$ is regarded as a node in the graph. Next, the KNN algorithm is used to find the neighbors of the nodes, and an association graph is constructed from the above dataset. The number of nodes in the graph is τ. According to the KNN algorithm, the $x_{i}$ nearest neighbors of the $k^{\prime }$ node can be represented as

(2) $$Ne(x_{i})=KNN(k^{\prime },x_{i},\psi ),$$

where $Ne\left(x_{i}\right)$ represents the set of neighbors of the $x_{i}$ sample, $KNN\left(\cdot \right)$ represents the output of the KNN algorithm, k′ represents the number of nearest neighbors given in the KNN algorithm, and $\psi =\left(x_{i+1},x_{i+2},\ldots ,x_{i+m}\right)$ represents the subset with mmm samples.

Finally, for the constructed KNN Graph, weights are assigned to the edges between nodes. In this paper, a Gaussian kernel weighting function is chosen, defined as follows:

(3) $$a_{ij}=\left\{\begin{array}{ll} \exp\left(-\frac{{{\left\|x_{i},x_{j}\right\|}_{2}}^{2}}{2\zeta^{2}}\right), & x_{j}\in Ne(x_{i}),\\ 0, & \mathrm{otherwise}, \end{array}\right.$$

where $a_{ij}$ represents the edge weight between nodes $x_{i}$ and $x_{j}$ in the KNN graph, ${\left\|\cdot \right\|}_{2}$ represents the Euclidean norm, and ζ represents the bandwidth of the Gaussian kernel, controlling the radial range of influence.

### 2.2. Legendre Graph Convolutional Filter

Based on the above content, one-dimensional vibration signal data with a non-Euclidean structure can be converted into weighted undirected graph structure data. For a given undirected graph:

(4) $$G=\left(V,E,A\right),$$

where $V=\left\{x_{1},x_{2},x_{3},\ldots ,x_{n}\right\}$ represents the set of vertices of the undirected graph G, $E=\left\{\langle x_{i},x_{j}\rangle \left|i,j\in \left\{1,2,3,\ldots ,n\right\}\right.\right\}$ represents the set of edges of the undirected graph G, $\langle x_{i},x_{j}\rangle$ represents the existence of an edge between nodes $x_{i}$ and $x_{j}$, $A=\left\{a_{ij}\right\}$ represents the weighted adjacency matrix of the undirected graph G, and $a_{ij}$ represents the weight of the edge between nodes $x_{i}$ and $x_{j}$.

According to the definition of the graph Laplacian matrix L,

(5) L=D−A,

where D represents the degree matrix of the weighted undirected graph G. For any given vertex $x_{i}$, its degree is represented as the weighted sum of all edges connected to that node, defined as follows:

(6) $$d_{i}=\sum_{j=1}^{n}a_{ij}.$$

The degree matrix D is represented as $D=\mathrm{diag}\left(d_{1},d_{2},d_{3},\ldots ,d_{n}\right)$, where D is an n×n diagonal matrix. Since the constructed graph is a weighted undirected graph, the adjacency matrix A is a symmetric matrix. Therefore, the Laplacian matrix L is a real symmetric matrix, which can be orthogonally diagonalized. In other words,

(7) $$\begin{array}{cc} L & =\left(\begin{array}{cccc} u_{1}\left(1\right) & u_{2}\left(1\right) & \cdots & u_{n}\left(1\right)\\ u_{1}\left(2\right) & u_{2}\left(2\right) & \cdots & u_{n}\left(2\right)\\ \vdots & \vdots & \vdots & \vdots \\ u_{1}\left(n\right) & u_{2}\left(n\right) & \cdots & u_{n}\left(n\right) \end{array}\right)\left(\begin{array}{cccc} \lambda_{1} & 0 & \cdots & 0\\ 0 & \lambda_{2} & \cdots & 0\\ \vdots & \vdots & \ddots & \vdots \\ 0 & 0 & \cdots & \lambda_{n} \end{array}\right){\left(\begin{array}{cccc} u_{1}\left(1\right) & u_{2}\left(1\right) & \cdots & u_{n}\left(1\right)\\ u_{1}\left(2\right) & u_{2}\left(2\right) & \cdots & u_{n}\left(2\right)\\ \vdots & \vdots & \vdots & \vdots \\ u_{1}\left(n\right) & u_{2}\left(n\right) & \cdots & u_{n}\left(n\right) \end{array}\right)}^{-1}\\ & =U\Lambda U^{-1}, \end{array}$$

where $\lambda_{i}$ represents the i eigenvalue of the Laplacian matrix L, $u_{i}\left(j\right)$ represents the j component of the i eigenvector corresponding to $\lambda_{i}$, and U is an orthogonal matrix, i.e., ${UU}^{T}={UU}^{-1}=I$. Therefore, $L={U\Lambda U}^{-1}={U\Lambda U}^{T}.$

Next, we define the Fourier transform on the graph. The traditional Fourier transform is known as

(8) $$F\left(\omega \right)=F\left[f\left(t\right)\right]={\int f\left(t\right)e}^{-i\omega t}dt,$$

which represents the transformation of the continuous signal $f\left(t\right)$ from the time domain to the frequency domain. It is known that $e^{-i\omega t}$ is the eigenfunction of the Laplacian operator. The Laplacian operator in continuous space corresponds to the Laplace matrix in discrete space, and the eigenvectors of the Laplace matrix form a set of orthogonal bases in N-dimensional space. According to reference [30], when extending the traditional Fourier transform to the graph Fourier transform, the eigenfunctions of the Laplacian operator are transformed into the eigenvectors of the Laplace matrix on the graph. The specific form is expressed as follows:

(9) $$F\left[f\left(t\right)\right]=\int_{-\infty }^{+\infty }f\left(t\right)e^{-i\omega t}dt\Rightarrow \hat{f}\left(\lambda_{l}\right)=F\left[f\left(i\right)\right]=\sum_{i=1}^{N}f\left(i\right)u_{l}\left(i\right).$$

It is known that the Fourier transform on the graph converts the graph signal from the vertex domain $f\left(i\right)$ to the spectral domain (corresponding to the eigenvectors of the Laplace matrix). Further expanding to matrix form, we obtain

(10) $$\left(\begin{array}{c} \hat{f}\left(\lambda_{1}\right)\\ \hat{f}\left(\lambda_{2}\right)\\ \vdots \\ \hat{f}\left(\lambda_{n}\right) \end{array}\right)=\left(\begin{array}{cccc} u_{1}\left(1\right) & u_{1}\left(2\right) & \cdots & u_{1}\left(n\right)\\ u_{2}\left(1\right) & u_{2}\left(2\right) & \cdots & u_{2}\left(n\right)\\ \vdots & \vdots & \vdots & \vdots \\ u_{n}\left(1\right) & u_{n}\left(2\right) & \cdots & u_{n}\left(n\right) \end{array}\right)\left(\begin{array}{c} f\left(1\right)\\ f\left(2\right)\\ \vdots \\ f\left(n\right) \end{array}\right).$$

That is, the graph Fourier transform is defined as

(11) $$\hat{f}=U^{T}f.$$

Similarly, the inverse graph Fourier transform in matrix form is given by

(12) $$\left(\begin{array}{c} f\left(1\right)\\ f\left(2\right)\\ \vdots \\ f\left(n\right) \end{array}\right)=\left(\begin{array}{cccc} u_{1}\left(1\right) & u_{2}\left(1\right) & \cdots & u_{n}\left(1\right)\\ u_{1}\left(2\right) & u_{2}\left(2\right) & \cdots & u_{n}\left(2\right)\\ \vdots & \vdots & \vdots & \vdots \\ u_{1}\left(n\right) & u_{2}\left(n\right) & \cdots & u_{n}\left(n\right) \end{array}\right)\left(\begin{array}{c} \hat{f}\left(\lambda_{1}\right)\\ \hat{f}\left(\lambda_{2}\right)\\ \vdots \\ \hat{f}\left(\lambda_{n}\right) \end{array}\right).$$

That is, the inverse graph Fourier transform is defined as

(13) $$f=U\hat{f}$$

It is known that the convolution theorem states that the Fourier transform of the convolution of two functions is equal to the product of their Fourier transforms. Extending this to the graph Fourier transform, we obtain

(14) $$F\left[f\left(i\right){*}_{G}h\left(i\right)\right]=F\left[f\left(i\right)\right]\times F\left[h\left(i\right)\right]=\hat{f}\hat{h},$$

where ${*}_{G}$ represents the graph convolution operator, $f\left(i\right)$ and $h\left(i\right)$ are the graph signals in the vertex domain, and $\hat{f}$ and $\hat{h}$ are the corresponding results after the graph Fourier transform. Similar to Equations (9) and (11), we obtain

(15) $$\hat{h}\left(\lambda_{l}\right)=F\left[h\left(i\right)\right]=\sum_{i=1}^{N}h\left(i\right)u_{l}\left(i\right)$$

The corresponding matrix form is

(16) $$\hat{h}=U^{T}h.$$

Assuming h is the convolution kernel, using Equation (14), we obtain

(17) $$F\left[f{*}_{G}h\right]=\left(\begin{array}{c} \hat{f}\left(\lambda_{1}\right)\hat{h}\left(\lambda_{1}\right)\\ \hat{f}\left(\lambda_{2}\right)\hat{h}\left(\lambda_{2}\right)\\ \vdots \\ \hat{f}\left(\lambda_{n}\right)\hat{h}\left(\lambda_{n}\right) \end{array}\right)=\left(\begin{array}{ccc} \hat{h}\left(\lambda_{1}\right) & & \\ & \ddots & \\ & & \hat{h}\left(\lambda_{n}\right) \end{array}\right)\left(\begin{array}{c} \hat{f}\left(\lambda_{1}\right)\\ \vdots \\ \hat{f}\left(\lambda_{n}\right) \end{array}\right)=\left(\begin{array}{ccc} \hat{h}\left(\lambda_{1}\right) & & \\ & \ddots & \\ & & \hat{h}\left(\lambda_{n}\right) \end{array}\right)U^{T}f,$$

where the first equality is the matrix form of Equation (14), the second equality uses the multiplication property of diagonal matrices, and the third equality uses Equation (11). Combining Equation (13) and using the convolution theorem, the inverse Fourier transform of Equation (17) gives the graph convolution result of the two as follows:

(18) $$\left(f{*}_{G}h\right)=U\left(\left(\begin{array}{ccc} \hat{h}\left(\lambda_{1}\right) & & \\ & \ddots & \\ & & \hat{h}\left(\lambda_{n}\right) \end{array}\right)U^{T}f\right)=U\left(\left(U^{T}h\right)⊙\left(U^{T}f\right)\right),$$

where ⊙ denotes the Hadamard product. In graph convolutional neural networks, when the graph signal x is acted upon by the convolution kernel $g_{\theta }\left(\Lambda \right)$, the above equation is also written as

(19) $$y=Ug_{\theta }\left(\Lambda \right)U^{T}x.$$

To avoid eigenvalue decomposition and reduce the computational complexity of graph convolution operations, an k-th order orthogonal polynomial is generally used to approximate the convolution kernel $g_{\theta }\left(\Lambda \right)$. This study proposes a novel graph convolution filter—Legendre orthogonal polynomials—to replace the traditional Chebyshev orthogonal polynomials. The specific expression is as follows:

(20) $$g_{\theta }(\Lambda )\approx \sum_{k=0}^{K}\theta_{k}P_{k}(\tilde{\Lambda }),$$

where $\theta_{k}$ represents the coefficient of the k-th order Legendre orthogonal polynomial, and $P_{k}$ represents the k-th order Legendre orthogonal polynomial. Since the range of the independent variable of Legendre polynomials is $\left[-1,1\right]$, the eigenvalue diagonal matrix Λ must be scaled to satisfy the range of the independent variable of Legendre polynomials. The specific operation is similar to the traditional Chebyshev orthogonal polynomial approximation convolution kernel. First, methods such as the power iteration method can be used to find the maximum eigenvalue $\lambda_{\max}$; then, the eigenvalues are normalized. Using the transformation $\tilde{\Lambda }=\frac{2\Lambda }{\lambda_{\max}}-I_{n}$, the eigenvalues $\tilde{\Lambda }\in \left[-1,1\right]$. Next, it is proven that Legendre orthogonal polynomials can be used to approximate the convolution kernel $g_{\theta }\left(\Lambda \right)$. Substituting Equation (20) into Equation (19), we obtain

(21) $$y=Ug_{\theta }\left(\Lambda \right)U^{T}x=U\left(\sum_{k=0}^{K}\theta_{k}P_{k}(\tilde{\Lambda })\right)U^{T}x.$$

Similar to the Chebyshev orthogonal polynomial approximation, we derive

(22) $$y=U\left(\sum_{k=0}^{K}\theta_{k}P_{k}(\tilde{\Lambda })\right)U^{T}x=\sum_{k=0}^{K}\theta_{k}P_{k}(U\tilde{\Lambda }U^{T})x=\sum_{k=0}^{K}\theta_{k}P_{k}(\tilde{L})x,$$

where, $\tilde{L}=\frac{2L}{\lambda_{\max}}-I_{n}$.

### 2.3. Graph Pooling

Similar to CNN on images, pooling operations can be defined on graphs to reduce the dimensions of the graph after the GCN layer. Diffpool [31] is a pooling method based on algebraic multigrid, which introduces a learnable hierarchical clustering module by training the matrix $S^{l}$ assigned to each layer:

(23) $$\left\{\begin{array}{c} Z^{l}=GNN_{l,embed}(A^{l},X^{l}),\\ S^{l}=Soft\max(GNN_{l,pool}(A^{l},X^{l})),\\ A^{l+1}={\left(S^{l}\right)}^{T}A^{l}S^{l}, \end{array}\right.$$

where $X^{l}$ is the node feature matrix, $A^{l}$ is the coarsened adjacency matrix of the l-th layer, and $S^{l}$ represents the probability that the nodes of the l-th layer can be assigned to the coarsened nodes of the l+1-th layer.

Although DiffPool achieves good results, it has a significant drawback: even if the graph itself is a sparse graph, the resulting $S^{l}$ is still a dense matrix. Top-K pool [32] overcomes this drawback by learning a projection vector $\vec{p}$, projecting the node features onto the vector $\vec{p}$ as the importance of the nodes, and retaining the Top-K nodes with the highest scores. The pooling graph (X′,A′) is calculated as follows:

(24) $$\left\{\begin{array}{c} X\prime ={\left(X⊙\tanh(\vec{z})\right)}_{\vec{i}},\\ A\prime =A_{\vec{i},\vec{i}},\\ \vec{i}=top-k(\vec{z},k)=X\cdot \vec{p}/{\left\|\vec{p}\right\|}_{2}, \end{array}\right.$$

where ${\left\|\cdot \right\|}_{2}$ is the L2 norm, k∈(0,1] represents the pooling ratio, $\cdot_{\vec{i}}$ is an indexing operation that obtains the slice at the specified index i, and top−k(⋅) represents the Top-K ranking mechanism. 

**Figure 2:**
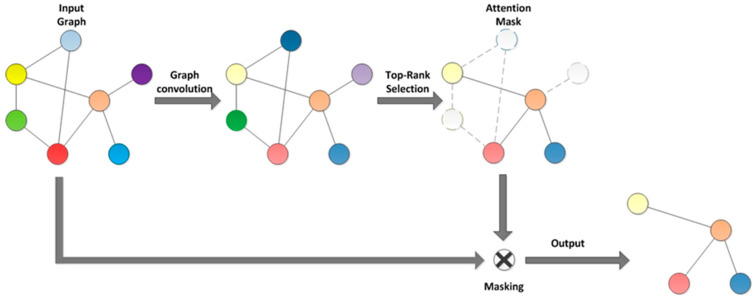
Self-attention graph pooling.

In traditional Top-K pooling, the selection of nodes is based on some metric of node features (such as the norm of feature vectors), retaining the most important K nodes. Although this method is simple and efficient, it may overlook global information in the graph structure and the interrelationships between nodes. This paper proposes a self-attention graph pooling strategy for fault diagnosis of rotating machinery (as shown in Figure 2). This strategy dynamically determines the importance of each node through a self-attention mechanism, improving the accuracy of node selection. This makes the pooling process better suited to the complexity and diversity of graph data, thereby enhancing the performance of graph neural networks in rotating machinery fault diagnosis. The steps of SAGP are as follows:

1. Node representation learning: First, use Legendre graph convolution to update the feature representation Z of each node. For each node v in the graph, its updated feature representation is $h_{v}$.
2. Attention score calculation: Calculate the attention score $a_{v}$ for each node.
3. Node selection and pooling: Based on the calculated attention scores, select the most important nodes to retain while removing nodes with lower scores. This process can be accomplished by directly selecting the Top-K nodes based on their scores.
4. Constructing the pooled graph: Construct the pooled graph based on the retained nodes and the edge connections from the original graph. This process may also include re-connecting edges or adjusting weights to maintain the coherence and completeness of the graph structure.

## 3. Experimental Section

To verify the effectiveness of the SA-LGCN model in rotating machinery fault diagnosis, experiments were performed using a dataset of planetary gearboxes measured in the laboratory. The experimental setup and process are described in the following subsections.

The programming language used in this study was Python 3.6, and the framework for the graph neural network algorithm was PyTorch Geometric 2.0.3. The computer utilized for the experiments featured a Core i7-12700 CPU @ 4.8 GHz and runs on a Windows 64-bit operating system. To improve training speed, an RTX 3070 GPU with 8 GB of memory was employed.

### 3.1. Dataset Introduction

**Figure 3:**
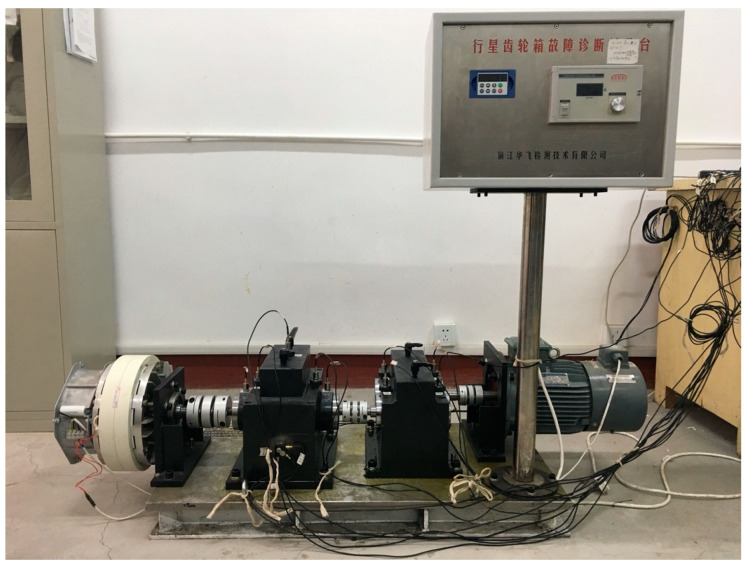
Planetary gearbox experimental platform.

The main components of the HFXZ-I planetary gearbox fault diagnosis experimental platform are illustrated in Figure 3. The platform comprises of a variable speed drive motor, bearings, a helical gearbox, a planetary gearbox, a magnetic powder brake, a variable-frequency drive controller, and a load controller.

**Figure 4:**
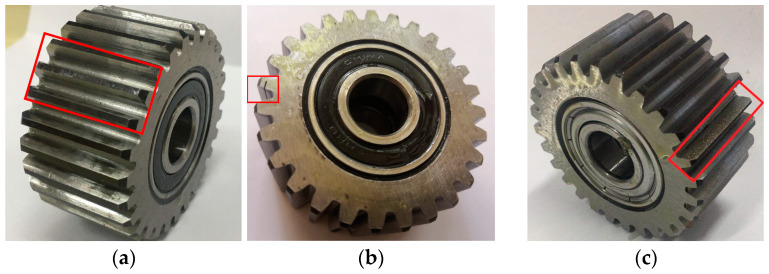

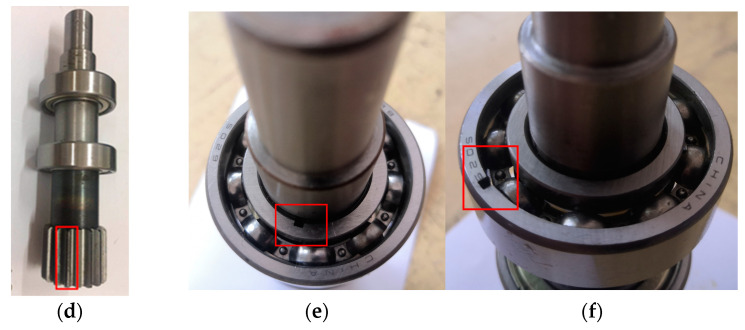
Common gearbox fault types: (**a**) gear pitting; (**b**) gear cracks; (**c**) gear wear; (**d**) sun gear broken teeth; (**e**) inner race defects; (**f**) outer race defects. Fault details are shown in the red boxes in the figure.

Using the planetary gearbox fault diagnosis experimental platform illustrated in Figure 3, various conditions were simulated. This platform includes common internal gearbox fault types such as gear pitting, gear cracks, gear wear (levels 1–3), sun gear broken teeth (levels 1–2), inner race defects, and outer race defects. The specific details of these faults are illustrated in Figure 4. Vibration signals were recorded using an accelerometer mounted on the top surface of the gearbox housing, with a sampling frequency of 10240 Hz. Continuous sampling was performed for 60 s under three motor speeds (20 Hz, 30 Hz, 50 Hz) and two load conditions (0.3 A, 0.5 A). Experiments were conducted for each failure mode to further verify the method’s generality and its ability to assess failure severity. The experiments were conducted 50 times, covering 10 fault types under 5 different load and speed conditions. It is worth noting that this experimental dataset includes varying degrees of faults, which further demonstrates the method’s decoupling ability and generality in feature extraction. The details of the data obtained from the experiments are presented in Table 1.

### 3.2. Data Preprocessing

First, the collected vibration signals from the ten conditions were normalized. Using the method described in Section 3.1, 1000 sub-samples were obtained, each with a length of 1024, and 100 graphs were constructed, each containing 10 nodes. The data were split into a training set and a test set in a ratio of 8:2.

### 3.3. Model Parameter Settings

The network structure and parameter settings of the SA-LGCN model are presented in Table 2. The hyper-parameters are set as follows: the model is trained using the Adam-optimized weighted loss function, with a momentum value of 0.9 and a batch size of 64; the input graph size is 10 × 1024 × 1024; and there are 200 training iterations. The initial learning rate was set to 0.01, with a decay of 1 × 10^−5^ after each epoch. It is worth noting that the above hyper-parameters were determined based on model performance. To ensure fairness, all comparative experiments were conducted in the same experimental environment.

### 3.4. Experimental Results and Analysis

To verify the effectiveness of SA-LGCN in rotating machinery fault diagnosis, multiple experiments were conducted using datasets with different loads and speeds: 20 Hz + 0.3 A, 20 Hz + 0.5 A, 30 Hz + 0.3 A, 30 Hz + 0.5 A, and 50 Hz + 0.5 A. The fault diagnosis accuracy of SA-LGCN was compared with that of five graph neural network models (ChebyNet, GCN, GAT, NCGCN, HGCN-LSL) and one deep learning model (CNN).

The comparison results of fault diagnosis accuracy across different models on five datasets are presented in Table 3. As shown in Table 3, the fault diagnosis accuracy of the SA-LGCN model is significantly higher than that of other models across various datasets, especially under high-load and high-speed conditions. This indicates that the SA-LGCN model more effectively captures the features in the vibration signals, thereby improving the accuracy of fault diagnosis. ChebyNet uses Chebyshev polynomials for spectral filtering, which improves accuracy to a certain extent but still has shortcomings in stability and computational efficiency. GCN’s excessive approximation of computational parameters results in the lowest accuracy under various conditions. GAT introduces an attention mechanism, which improves fault diagnosis accuracy but increases computational complexity. CNN performs fairly well in handling vibration signals, but since it can only process Euclidean space data, it performs worse than graph neural networks on complex signals and graph-structured data. Compared to two state-of-the-art GCN models, the proposed model improves accuracy by 4.76% to 8.79% over NHGCN and by 5.71% to 9.88% over HGCN-LSL.

**Figure 5:**
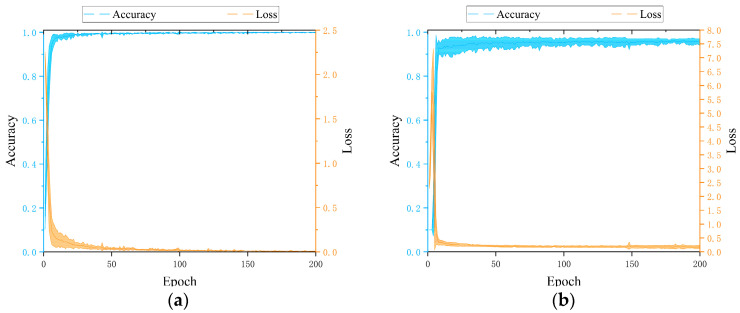
Training process of SA-LGCN: (**a**) the loss and accuracy of the training set; (**b**) the loss and accuracy of the validation set.

Next, the training process of the proposed model under the condition of 50 Hz + 0.5 A is visualized. The loss and accuracy of the training set are presented in Figure 5a, while the loss and accuracy of the validation set are presented in Figure 5b. From the loss and accuracy curves on the training and validation sets, it can be observed that the SA-LGCN model converges quickly during training, with accuracy rapidly increasing and loss values rapidly decreasing, indicating good convergence of the model. After about 10 epochs, the accuracy and loss values on both the training and validation sets tend to stabilize, indicating stable performance of the model on both sets without obvious overfitting or underfitting.

**Figure 6:**
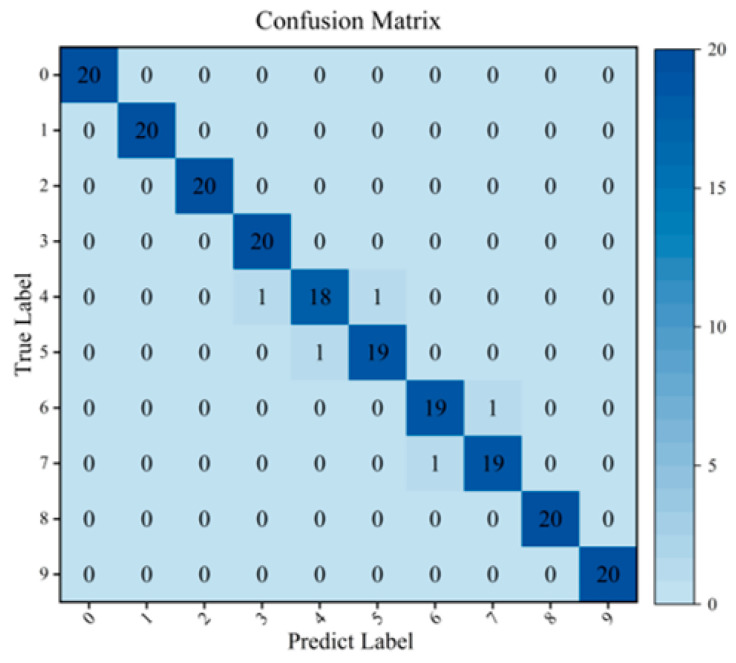
Confusion matrix results on SA-LGCN.

Additionally, the confusion matrix of the SA-LGCN model under the 50 Hz + 0.5 A condition is presented in Figure 6, where the X-axis and Y-axis represent the predicted labels and the true labels, respectively. The results show that the SA-LGCN model can effectively identify normal conditions and six different fault conditions at various locations. The model can also distinguish between different levels of faults fairly well. Out of 200 test samples, five samples were misclassified. Specifically, two samples with label 4 were predicted as labels 3 and 5, one sample with label 5 was predicted as label 4, one sample with label 6 was predicted as label 7, and one sample with label 7 was predicted as label 6. This is because labels 3, 4, and 5 correspond to three different levels of gear wear, and labels 6 and 7 correspond to two levels of sun gear broken teeth. The fault features among these labels are very similar.

**Figure 7:**
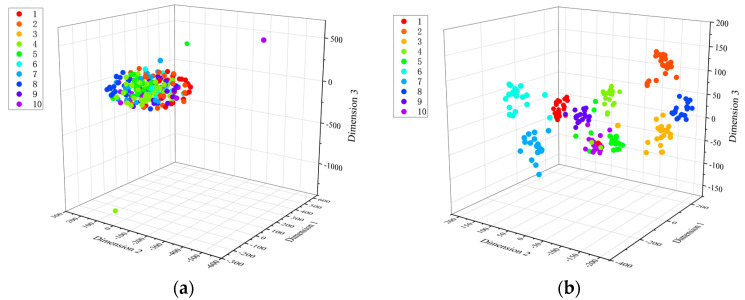
SA−LGCN feature visualization: (**a**) input feature; (**b**) output feature.

To verify the proposed model’s ability to learn discriminative features further, t-SNE was used to project the features learned by the SA-LGCN model in the fully connected layer from high-dimensional space to three-dimensional space for visualization. The three-dimensional scatter plot illustrates the change in sample distribution during the fault diagnosis process. Each point in the figure represents a graph sample, with different colors representing different health states. As illustrated in the figure, the feature distribution of the original graph is highly clustered (Figure 7a). After applying the proposed method (Figure 7b), the sample distribution within each category becomes more concentrated, while the distribution between categories becomes more discrete. Clearly, the proposed method demonstrates strong effectiveness in the fault diagnosis of rotating machinery.

### 3.5. Ablation Study

To verify the effectiveness of each component in the SA-LGCN model further, three sets of ablation experiments were designed, where key parts of the model were removed or replaced to observe the impact on performance. This approach allows for a clear determination of the contribution of each component to the overall performance of the model.

#### 3.5.1. Experimental Setup

Baseline model: The complete SA-LGCN model, including the Legendre-polynomial-based fast local spectral filter and the self-attention graph pooling method.

Ablation 1 (without Legendre filter): The Legendre-polynomial-based fast local spectral filter is removed and replaced with the traditional Chebyshev filter.

Ablation 2 (without self-Attention graph pooling): Self-attention graph pooling is removed and replaced with the Top-K pooling.

Ablation 3 (without Legendre filter and self-attention graph pooling): Both the Legendre-polynomial-based fast local spectral filter and self-attention graph pooling are removed and replaced with the traditional Chebyshev filter and Top-K pooling.

#### 3.5.2. Ablation Experiment Results

Experiments were conducted on the above models using the same datasets (20 Hz + 0.3 A, 20 Hz + 0.5 A, 30 Hz + 0.3 A, 30 Hz + 0.5 A, 50 Hz + 0.5 A), and the fault diagnosis accuracy of each model was recorded. The results of the ablation experiments are presented in Table 4.

As shown in Table 4, the accuracy of the proposed model improves by 2.35% to 9.70% compared to Ablation 1, by 1.56% to 7.32% compared to Ablation 2, and by 8.37% to 14.38% compared to Ablation 3. These significant improvements demonstrate that the model achieves strong fault diagnosis performance. Compared to the Chebyshev-based fast local spectral filter, the Legendre-polynomial-based fast local spectral filter plays an important role in improving the model’s stability. Compared to Top-K pooling, the graph pooling based on the self-attention mechanism more effectively focuses on key nodes, thereby improving the accuracy of fault diagnosis.

## 4. Conclusions

This paper proposes a Legendre graph convolutional network with a self-attention graph pooling method, applied to the fault diagnosis of rotating machinery. The proposed method offers the following advantages: (1) converting one-dimensional vibration signals into association graphs in non-Euclidean space accurately characterizes fault information, simplifying the fault diagnosis process, eliminating the need for manual intervention, and achieving end-to-end multi-source information fusion and classification; (2) the self-attention graph pooling method enhances the accuracy of node selection, making the pooling process better suited to the complexity and diversity of graph data; (3) experimental results demonstrate that the proposed method effectively identifies different severities and types of faults in rotating machinery. Under various loads and speeds, the proposed method outperforms other models in diagnostic accuracy and load adaptability.

## Figures and Tables

**Table 1:** Details about the dataset.

Status Label	Fault Location	Fault Description	Motor Speed (Hz)	Load (A)
0	None	Normal	20/30/50	0.3/0.5
1	Gear	Gear pitting	20/30/50	0.3/0.5
2	Gear	Gear cracks	20/30/50	0.3/0.5
3	Gear	Gear wear (level 1)	20/30/50	0.3/0.5
4	Gear	Gear wear (level 2)	20/30/50	0.3/0.5
5	Gear	Gear wear (level 3)	20/30/50	0.3/0.5
6	Gear	Sun gear broken teeth (level 1)	20/30/50	0.3/0.5
7	Gear	Sun gear broken teeth (level 2)	20/30/50	0.3/0.5
8	Bearing	Inner race defects	20/30/50	0.3/0.5
9	Bearing	Outer race defects	20/30/50	0.3/0.5

**Table 2:** Structure parameters and hyper-parameters setup of SA-LGCN.

Structure	Outputs Size
Input	10 × 1024 × 1024
Legendre graph convolution 1	1024 × 1024
Self-attention pool 1	1024
Legendre graph convolution 2	1024 × 1024
Self-attention pool 2	1024
Legendre graph convolution 3	1024 × 1024
Self-attention pool 3	1024
Fc1	1024 × 512
Dropout	0.2
Fc2	512 × C
hyper-parameters	Optimizer: Adam the momentum of Adam = 0.9 Batch size = 64 Learning rate = 0.01 Learning rate decays = 1 × 10^−5^

**Table 3:** Accuracy of each model under five datasets.

Model	Accuracy				
	20 Hz + 0.3 A	20 Hz + 0.5 A	30 Hz + 0.3 A	30 Hz + 0.5 A	50 Hz + 0.5 A
ChebyNet [30]	93.50%	90.24%	89.43%	86.99%	96.00%
GCN [12]	64.23%	68.75%	56.91%	58.96%	61.79%
GAT [14]	91.87%	86.99%	90.24%	92.68%	86.18%
NCGCN [24]	92.25%	89.17%	90.36%	87.96%	91.37%
HGCN-LSL [29]	89.75%	91.85%	88.37%	90.71%	87.62%
CNN	78.05%	83.74%	84.55%	86.18%	88.62%
SA-LGCN	99.19%	97.56%	95.12%	96.75%	97.50%

**Table 4:** Accuracy of each ablation experiment.

Datasets	Baseline	ChebyNet + SAGP	LGCN + Top-K Pool	ChebyNet + Top-K Pool
20 Hz + 0.3 A	99.19%	96.56%	91.87%	85.87%
20 Hz + 0.5 A	97.56%	95.18%	95.75%	83.64%
30 Hz + 0.3 A	95.12%	92.37%	93.56%	86.75%
30 Hz + 0.5 A	96.75%	88.62%	94.93%	82.37%
50 Hz + 0.5 A	97.50%	87.80%	95.62%	84.40%

## Data Availability

The ownership belongs to corresponding author. Please contact jyhuang@nuc.edu.cn if necessary.
